# Resolvin D1 in the Lipopolysaccharide-Induced Inflammatory Microenvironment Mediates Resolution in Human Monocytic THP-1 Cells

**DOI:** 10.3390/biomedicines14051124

**Published:** 2026-05-15

**Authors:** Zhe Xing, Qian Zhao, Xiaoli He, Jiazheng Cai, Yaxin Xue, Christopher Graham Fenton, Alpdogan Kantarci, Kristin Andreassen Fenton, Xiaoli An, Ying Xue

**Affiliations:** 1 School/Hospital of Stomatology, Lanzhou University, Lanzhou 730000, China; 2 Key Laboratory of Dental Maxillofacial Reconstruction and Biological Intelligence Manufacturing, Lanzhou University, Lanzhou 730000, China; 3 Department of Medical Biology, Faculty of Health Sciences, UiT the Arctic University of Norway, 9037 Tromsø, Norway; 4 Department of Orthodontics and Dentofacial Orthopedics, The First Clinical Medical College, Lanzhou University, Lanzhou 730000, China; 5 School/Hospital of Stomatology, Xi’an Jiaotong University, Xi’an 710000, China; 6 Computational Biology Unit, Department of Informatics, University of Bergen, 5008 Bergen, Norway; 7 Genomics Support Centre Tromsø (GSCT), Department of Clinical Medicine, Faculty of Health Sciences, UiT the Arctic University of Norway, 9037 Tromsø, Norway; 8 Department of Developmental and Surgical Sciences, School of Dentistry, University of Minnesota, Minneapolis, MN 55455, USA; 9 School of Dental Medicine, Harvard University, Boston, MA 02115, USA; 10 Department of Clinical Dentistry, Faculty of Health Sciences, UiT the Arctic University of Norway, 9037 Tromsø, Norway

**Keywords:** LPS, resolvin D1, mRNA seq, MAPK, pro-resolving, THP-1 cells

## Abstract

**Objectives**: An infectious trigger can initiate a systemic inflammatory response, which in turn activates immune cells and causes the release of various mediators. Local mediators, such as resolvin D1 (RvD1), actively interact with immune cells to promote the resolution of inflammation. This study aimed to determine the impact of RvD1 on the inflammatory response mediated by monocytes in response to LPS. **Methods**: To investigate the mechanism by which RvD1 affects the monocyte-mediated inflammatory response to LPS, human THP-1 monocytic cells were treated with LPS, RvD1, or vehicle for 24 h. Inflammatory cytokines, interleukin-1β (IL-1β) and tumor necrosis factor (TNF-α), were measured using enzyme-linked immunosorbent assay (ELISA). RNA sequencing (RNA-seq) was used to identify differentially expressed genes (DEGs). The NF-κB and MAPK p38 signaling pathways were validated using real-time quantitative PCR (RT-qPCR) and Western blotting (WB). **Results**: RvD1 diminished the levels of IL-1β and TNF-α in LPS-induced inflammation. RvD1 significantly enhanced the mRNA expression of *CREB*, *NRF2*, and *BCL-2*. In addition, RvD1 significantly decreased the mRNA expression of *CASP3*. RvD1 regulated the inflammatory process in human monocytic THP-1 cells via the NF-κB p65 (MyD88, p65) and p38 MAPK signaling pathways (p38, BCL-2) and further suppressed the expression of apoptotic factors (PI3K, caspase-3). **Conclusions**: RvD1 has been shown to exert pro-resolving effects by regulating the anti-apoptotic gene BCL-2 and activating the NF-κB p65 and MAPK p38 signaling pathways.

## 1. Introduction

Inflammation is a complex biological process triggered in response to injury, trauma, infection or other danger signals and involves an acute inflammatory response that initiates inflammation and a pro-resolving immune response [1,2,3]. Lipopolysaccharide (LPS), which is found on the outer membrane of *E. coli* and other Gram-negative bacteria (GNB), is one of the most common inflammatory triggers [4,5,6]. Even at very low concentrations, LPS triggers an inflammatory cascade [7]. In vivo, LPS activates Toll-like receptor (TLR) signaling to trigger a cytokine-mediated inflammatory response and initiate host defense mechanisms [8]. The accumulation of LPS in peripheral blood activates neutrophils (Polymorphonuclear neutrophils, PMNs), monocytes, and macrophages to produce inflammatory cytokines such as interleukin-1 (IL-1β), tumor necrosis factor (TNF-α), and interleukin-6 (IL-6) [9]. At the systemic level, the activation of these inflammatory cytokines induces alterations in metabolic processes, hormonal balance, and the neuroendocrine system, ultimately resulting in abnormal cellular function and progressive dysfunction or failure of multiple organ systems.

Monocytes and macrophages act as the first line of defense as part of host defense, and both can rapidly infiltrate sites of tissue damage, contributing to a return to homeostasis [10]. In particular, the resolution of acute inflammation is initiated when the very first leukocytes arrive at the inflammatory site [11]. Resolvin D1 (RvD1), an endogenous pro-resolving lipid molecule, has demonstrated efficacy against live bacteria, promoting the resolution of several models of sepsis and lung injury and reducing the need for antibiotics in infected mice [12,13]. These results show that RvD1 effectively attenuates bacteria-induced inflammation, especially LPS-induced inflammation. There is evidence that RvD1 binds to neutrophils, Th1 cells, and Th17 cells mainly through cell-specific miRNA regulation, allowing the activation of different intracellular pathways [14]. Furthermore, recent studies have shown that RvD1 mainly activates intracellular signaling pathways through formyl peptide receptor 2 (ALX/FPR2) and cell-specific miRNA219 on the surface of monocytes and synergistically reduces the chemotaxis and infiltration of monocytes to promote the inflammation resolution process [15,16]. However, the mechanism of its specific activation of intracellular signaling pathways is still unclear. Thus, investigating the mechanism by which RvD1 regulates acute inflammation at the gene level is meaningful.

Most studies have used phorbol myristate acetate-activated (PMA) THP-1 monocytic cells as a model for macrophages to simulate the polarized activity of macrophages under inflammatory conditions and study the LPS-mediated actions related to macrophages [17]. However, previous studies have shown that undifferentiated THP-1 monocytic cells can still mediate the expression of Toll-like receptors under LPS stimulation [18,19,20]. Specifically, undifferentiated THP-1 cells can express TLRs upon LPS stimulation due to their intrinsic nature as a monocytic cell line, which harbors an intact and functional TLR signaling pathway. Moreover, resolvin D2 (RvD2) treatment decreases TLR4 expression to mediate resolution in human monocytes [21]. Therefore, in this study, THP-1 monocytic cells without PMA treatment were chosen as an in vitro cell model for resolvin-mediated inflammation resolution.

Based on the critical role of monocytes in inducing and maintaining inflammation, a human monocyte-centric model of acute inflammation was established. Understanding how RvD1 regulates inflammation-related genes to affect monocytes and uncovering the underlying mechanisms by which RvD1 regulates acute inflammation are the core of this research.

## 2. Materials and Methods

### 2.1. Cell Culture

Human monocytic THP-1 cells were obtained from ATCC (Manassas, VA, USA). RPMI 1640 (Basal Media, Shanghai, China) was used to maintain the cell lines with 10% fetal bovine serum (Abwbio, Shanghai, China), 100 µg/mL penicillin, 100 µg/mL streptomycin sulfate, and 200 mmol/L L-glutamine. The cell lines were cultured in a humidified atmosphere of 5% CO_2_ at 37 °C.

### 2.2. RvD1 Treatment and LPS Exposure

RvD1 (catalog # 10012554) was obtained from Cayman Chemicals (Ann Arbor, MI, USA). Lipopolysaccharide (LPS, *Escherichia coli* 0111: B4) was obtained from Sigma–Aldrich (St. Louis, MO, USA). Phosphate-buffered saline (PBS, B320JK) was obtained from Basal Media (Shanghai, China). The cells were divided into four groups: the vehicle-treated control group, the LPS group (100 ng/mL), the RvD1 group (100 ng/mL), and the LPS+RvD1 group (100 ng/mL). Different groups of supernatants were collected after 24 h and analyzed via ELISA (Supplementary Table S1). The concentrations of LPS and RvD1 used were based on our previous studies [22,23,24].

### 2.3. Enzyme-Linked Immunosorbent Assay (ELISA)

THP-1 cells (2 × 10^5^ cells/well) were seeded into 6-well plates and divided into four groups. The levels of secreted TNF-α (catalog # JL10208) and IL-1β (catalog # JL13662) in culture supernatants were determined with sandwich ELISA kits (Jianglai Biology, Shanghai, China). The standard solution, control solution, or sample solution was added to an ELISA plate precoated with a specific capture monoclonal antibody. Then, 100 µL of streptavidin-conjugated horseradish peroxidase was added to each well, followed by incubation of the mixture for 60 min at room temperature in the dark. After thorough washing, a substrate solution containing hydrogen peroxide and chromogen was added. After 15 min, a stop solution was added to each well. Cytokine levels were measured at 450 nm using a plate reader and normalized to the standard curve.

### 2.4. mRNA Sequencing

After LPS treatment, the cells were harvested, and total RNA was extracted with TRIzol reagent (AmbionÒ, Austin, TX, USA). The integrity and concentration of RNA were assessed, ensuring that the RNA integrity number (RIN) values were ≥6.5. Libraries for RNA sequencing were prepared via the TruSeq RNA Sample Preparation Kit V2 (Illumina, San Diego, CA, USA), with purified and fragmented mRNAs. Strand-specific libraries were constructed to increase the accuracy of gene function annotation and expression analysis. The libraries were subjected to PCR amplification and size selection of DNA fragments between 300 and 400 bp, followed by quality assessment using an Agilent 2100 Bioanalyzer (Agilent Technologies, Santa Clara, CA, USA). The libraries were sequenced on the Illumina Hiseq 2500 platform (Illumina, USA) utilizing a paired-end sequencing method with 2 × 150 bp, resulting in the acquisition of FastQ data.

### 2.5. Real-Time Quantitative Polymerase Chain Reaction

Total RNA was isolated using the TRIzol-chloroform extraction method (Ambion, Austin, TX, USA) and reverse transcribed into cDNA (Easy Quick RT MasterMix, CWBIO, Beijing, China). A thermal cycler (TC-96/G/HbC, BIOER, Hangzhou, China) was used for reverse transcription or PCR amplification. Real-time quantitative PCR (RT-qPCR) was performed on a Rotor-Gene Q5plex instrument (QIAGEN, Düsseldorf, Germany). *GAPDH* was used as a reference gene. Each sample was analyzed with the following primer sets listed in Supplementary Table S1. Data were analyzed using the comparative (2^−△△CT^) method [25].

### 2.6. Western Blotting

Total protein extraction buffer was prepared by adding 1% PMSF (1mM, Solarbio, Beijing, China) to RIPA solution (Solarbio, Beijing, China). After extraction, total protein concentration was determined using a BCA protein assay kit (CWBIO, China). A total of 40 µg of total protein was loaded onto the polyacrylamide (PAA) gel. Proteins from each group were separated by 12% SDS–PAGE and transferred to nitrocellulose membranes (Bio–Rad, Hercules, CA, USA). The membranes were blocked with 5% nonfat milk. Then, they were incubated with primary and secondary antibodies (1:3000, Proteintech, Rosemont, IL, USA) and visualized using a chemiluminescence system (ChemiScope 6100, QinXiang, Shanghai, China). NcmECL Ultra (NCM Biotech, Suzhou, China) was used to visualize protein bands using a chemiluminescence device (VILBER, Marne-la-vallée, France). The primary antibodies included nuclear factor-kappa B (NF-κB p65, 1:1000, Proteintech, USA), p38 mitogen-activated protein kinase (MAPK p38, 1:1000, Proteintech, USA), phosphoinositide 3-kinase (PI3K, 1:1000, Proteintech, USA), myeloid differentiation factor 88 (MyD88, 1:1000, Proteintech, USA), protein kinase B (AKT, 1:1000, Proteintech, USA), caspase-3 (CASP3, 1:1000, Proteintech, USA), B-cell lymphoma-2 (BCL-2, 1:1000, Proteintech, USA), and β-actin (1:1000, Proteintech, USA). Protein band densities were analyzed with ImageJ’s gel analysis tool (version 1.46r, Bethesda^®^, Montgomery, MD, USA).

### 2.7. Statistical Analysis

The data were obtained from three independent experiments and are reported as the means ± SDs (n = 3). For the mRNA sequencing data, differentially expressed genes (DEGs) were defined as an absolute value of log2 (fold change) > 1 and a *p*-value < 0.05. Other data were analyzed and visualized using GraphPad Prism software (version 10.3.1, San Diego, CA, USA). Data were assessed for normality and homogeneity of variance using the Shapiro–Wilk and Levene tests, respectively. For multiple comparisons, two-way analysis of variance (ANOVA) with Tukey’s post hoc test was used. A *p*-value < 0.05 was considered statistically significant unless otherwise indicated.

## 3. Results

### 3.1. Resolvin D1 Diminished LPS-Induced Inflammation in a Monocytic Cell Model

**Figure 1:**
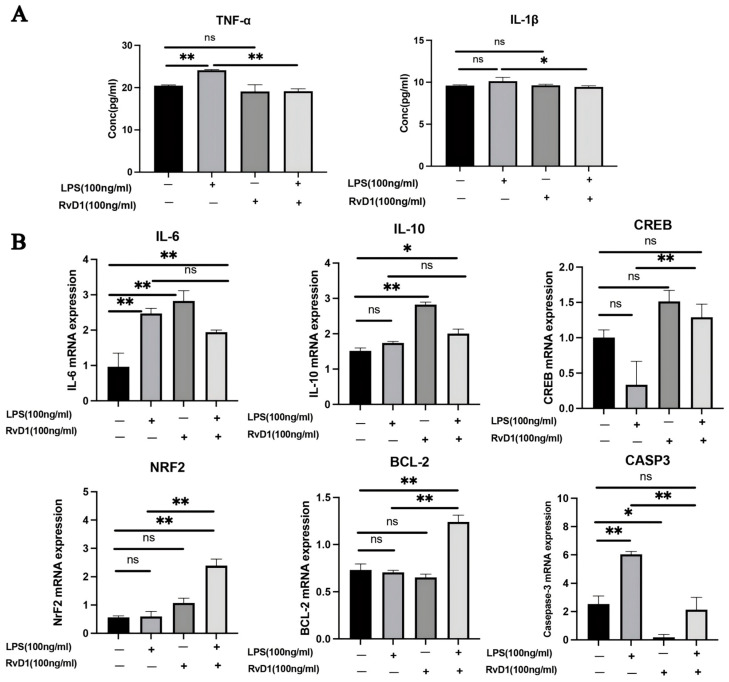
RvD1 modulates pro-inflammatory, anti-inflammatory, and apoptotic cytokine levels. (**A**) ELISA quantification of TNF-α and IL-1β levels in culture supernatants. (**B**) qPCR analysis of *IL-6*, *IL-10*, *CREB*, *NRF2*, *BCL-2*, and *CASP3* mRNA expression. (** *p* < 0.01; * *p* < 0.05; ns, not significant).

To address the role of RvD1 in an LPS-induced monocytic inflammation model, inflammatory and regulatory markers were assessed by ELISA and qPCR. ELISA analysis showed that LPS stimulation significantly increased TNF-α levels compared with the control group (*p* = 0.0035). Compared with the LPS-only group, RvD1 treatment significantly reduced the secretion of IL-1β and TNF-α (*p* = 0.0354 and *p* = 0.0005, respectively; Figure 1A).

qPCR analysis showed that LPS significantly increased the mRNA expression of *IL-6* and *CASP3* (*p* = 0.0006 for both; Figure 1B). Compared with the LPS-only group, LPS+RvD1 treatment showed a trend toward reduced *IL-6* expression and increased *IL-10* expression, although these changes did not reach statistical significance (*p* = 0.1218 and *p* = 0.1155, respectively). Notably, RvD1 significantly enhanced the mRNA expression of *CREB* (*p* = 0.0079) and *NRF2* (*p* < 0.0001), reduced *CASP3* expression (*p* = 0.0003), and increased *BCL-2* expression (*p* = 0.0017; Figure 1B). These results suggest that RvD1 suppresses LPS-induced inflammatory cytokine production and may promote cytoprotective and anti-apoptotic transcriptional responses in monocytic cells.

### 3.2. Screening and Enrichment Analyses of DEGs

**Figure 2:**
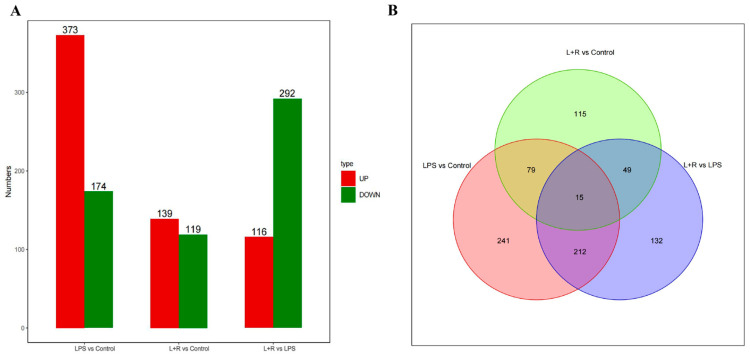
DEGs across different groups. (**A**) The number of DEGs in each group. (**B**) Distribution of DEGs in different groups. Downregulated (n = 174) and upregulated (n = 373) genes in the LPS vs. Control groups. Downregulated (n = 119) and upregulated (n = 139) genes in the L+R vs. Control groups. Downregulated (n = 292) and upregulated (n = 116) genes in the L+R vs. LPS groups.

Principal component analysis (PCA) was performed to visualize the gene expression profiles of three sample groups: the control (Control), LPS (LPS), and the LPS+RvD1 (L+R) groups. The PCA revealed distinct clustering patterns among these groups (Supplementary Figure S1). DEGs were subjected to statistical analysis to determine their counts and distribution across the comparison groups. The bar chart below illustrates the distribution of DEGs in each comparison group (Figure 2A). Compared with the control group, 373 DEGs were upregulated, and 174 DEGs were downregulated in the LPS group (Figure 2A). Compared with the control group, 139 DEGs were upregulated, and 119 DEGs were downregulated in the LPS+RvD1 group (Figure 2A). Interestingly, only 116 DEGs were upregulated, and 292 DEGs were downregulated in the LPS+RvD1 group compared with the LPS group (Figure 2A). Overlaps occurred among the three groups of DEGs (Figure 2B). To visualize these shared genes, the top 50 DEGs common to the three groups were plotted in a heatmap (Supplementary Figure S2).

Based on the distribution of DEGs among the three sample groups, the results are as follows: (1) a total of 187 common DEGs were upregulated in the LPS group vs. the Control group but downregulated in the LPS+RvD1 group vs. the LPS group (Figure 2B) (Table 1). (2) 40 common DEGs were downregulated in the LPS group vs. the Control group but upregulated in the LPS+RvD1 group vs. the LPS group (Figure 2B) (Table 1). (3) A total of 309 DEGs were commonly upregulated in the LPS group vs. the Control group but were inhibited by RvD1 treatment in the LPS+RvD1 group vs. the Control group (Figure 2B) (Table 2). (4) Of these 309 DEGs, only two DEGs were downregulated in the LPS+RvD1 group vs. the Control group, while the remaining 307 DEGs exhibited negligible expression in the LPS+RvD1 group vs. the Control group (Figure 2B) (Table 2). The top 20 genes were selected from these common DEGs based on the absolute value of log2 fold change. Through differential GO enrichment and hierarchical clustering analysis, we identified certain DEGs that are associated with inflammation. The expression levels of CREB1 and BCL-2L12 were downregulated by LPS but upregulated by RvD1 (Table 3). Additionally, the expression levels of DEGs such as IL1B, TNFAIP6, CASP3, and NFKB2 were upregulated by LPS but suppressed by RvD1 treatment (Table 3).

### 3.3. Resolvin D1 Regulates the Monocyte-Mediated Inflammatory Response to LPS: GO and KEGG Pathways Analysis

**Figure 3:**
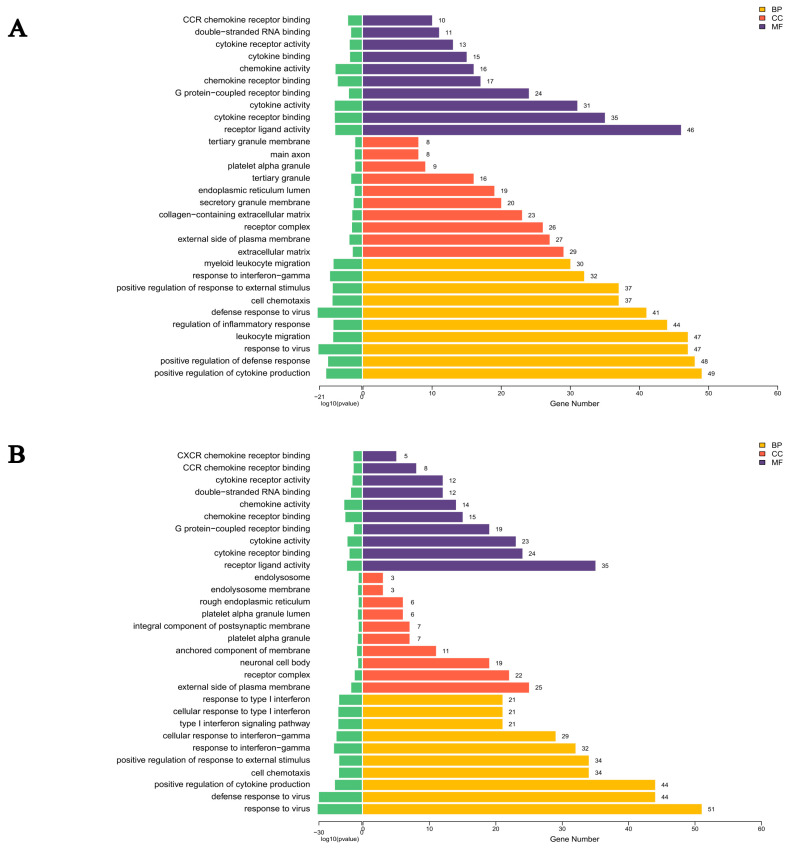
GO enrichment bar chart: LPS group vs. Control group and the LPS+RvD1 group vs. the LPS group. (**A**) Differential gene GO enrichment bar chart: LPS group vs. Control group. It reflects the distribution of the number of target genes in the enriched GO function. (**B**) Differential gene GO enrichment bar chart: LPS+RvD1 group vs. LPS group.

**Figure 4:**
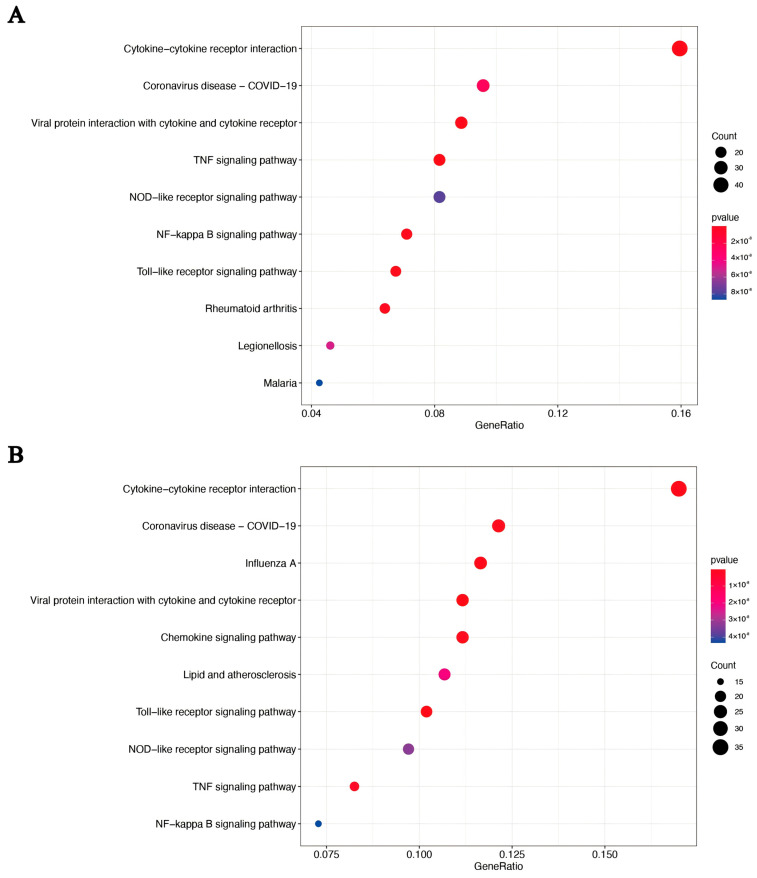
KEGG enrichment scatterplot: LPS group vs. Control group and the LPS+RvD1 group vs. the LPS group. (**A**) Differential gene KEGG enrichment scatterplot: LPS group vs. Control group. (**B**) Differential gene KEGG enrichment scatterplot: LPS+RvD1 group vs. LPS group.

Based on Gene Ontology (GO) functional annotation, the significant DEGs were classified according to biological processes (BP), cellular components (CC), and molecular functions (MF) (Figure 3A,B). KEGG enrichment analysis was conducted to identify the top 10 differentially expressed mRNAs associated with cellular signaling pathways. In both the LPS group and LPS+RvD1 group, the inflammation-related signaling pathways common to both groups included the TNF signaling pathway, NOD-like receptor signaling pathway, NF-κB signaling pathway, and Toll-like receptor signaling pathway (Figure 4A,B). Moreover, transcriptome analysis revealed that the LPS group, compared to the control group, showed upregulation of genes associated with inflammatory signaling pathways, specifically the MAPK and NF-κB p65 signaling pathways (Supplementary Figure S3). Notably, transcriptomic analysis indicated that the treatment groups (LPS group and LPS+RvD1 group) displayed the key genes of the MAPK p38 pathway and its downstream signaling cascades and influenced the expression of apoptosis-related genes.

### 3.4. Effects of Resolvin D1 on MAPK in LPS-Induced THP-1 Cells

**Figure 5:**
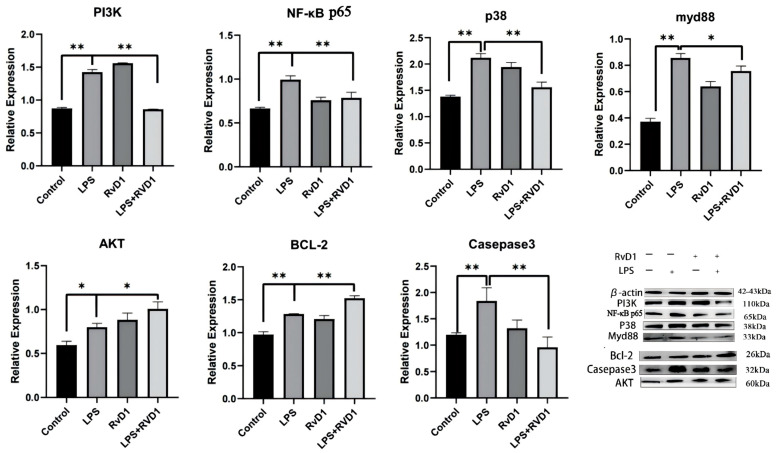
Effects of resolvin D1 on MAPK signaling in LPS-treated THP-1 cells. RvD1 decreased the protein expression of PI3K, CASP3, MyD88, NF-κB p65 and MAPK p38 and increased the protein expression of the anti-apoptotic factors BCL-2 and AKT. THP-1 cells were treated with RvD1 (100 ng/mL) and LPS (100 ng/mL) for 24 h (** *p* < 0.01; * *p* < 0.05).

RvD1 attenuated LPS-induced expression of the MAPK p38 signaling pathway. In THP-1 cells, RvD1 suppressed PI3K expression and reduced the levels of NF-κB p65 and MyD88 (Figure 5). RvD1 increased BCL-2 gene and protein expression, as confirmed by WB and RT-PCR. Additionally, the expression of the CASP3 gene and protein was significantly upregulated following RvD1 treatment (Figure 1B and Figure 5).

## 4. Discussion

Inflammation resolution is recognized as an active biochemical process in which mediators such as cyclooxygenase-2 (COX-2) lead to prostaglandin E2 (PGE2) production, shifting neutrophil lipid mediator biosynthesis from the 5-lipoxygenase (5-LOX) and leukotriene B4 (LTB4) pathways to the pro-resolving lipoxin A4 (LXA4) pathway [26,27,28]. This shift enhances monocyte migration and phagocytosis of apoptotic neutrophils [11]. RvD1 plays a crucial role in this process by modulating immune cell function through the regulation of miRNAs, particularly *miR-146b*, *miR-21*, *miR-219*, and *miR-208a*, which target pathways such as 5-LOX, reducing LTB4 synthesis and further recruitment of PMNs [15,29,30]. This regulation is time-specific, with significant miRNA expression changes peaking at 24 h [11,15]. Understanding the impact of RvD1 on inflammation at the genetic level through high-throughput mRNA sequencing could advance therapeutic strategies for inflammatory diseases [31,32,33,34].

In this study, we demonstrated that RvD1 effectively inhibits LPS-induced pro-inflammatory cytokine production in human monocytes by regulating gene expression. Using mRNA sequencing, we identified genes that were differentially expressed across the control, LPS, and LPS+RvD1 groups. Our analysis revealed that 187 genes upregulated by LPS were downregulated following RvD1 treatment, whereas 40 genes downregulated by LPS were upregulated in the LPS+RvD1 group (Table 1). These findings suggest targeted modulation by RvD1 at the genetic level, particularly with respect to the regulation of the *BCL-2* gene, which was significantly downregulated in the LPS group and upregulated in the LPS+RvD1 group (Figure 1B). Moreover, some DEGs that were upregulated in the LPS group relative to control were suppressed by RvD1 treatment in the LPS+RvD1 group (Table 2). Additionally, we observed that RvD1 treatment attenuated the upregulation of 309 genes induced by LPS, with only 64 of these genes still upregulated in the LPS+RvD1 group, indicating a robust inhibitory effect of RvD1 on LPS-induced gene expression. This gene regulation pattern underscores the potential mechanisms by which RvD1 mediates its anti-inflammatory effects. Further investigations into these specific genetic pathways are warranted to better understand and enhance the therapeutic application of RvD1 under inflammatory conditions.

Through differential GO enrichment and hierarchical clustering analysis, we identified key genes and signaling pathways, including *CREB1* and *BCL-2L12*, which were downregulated by LPS but upregulated by RvD1 (Table 3). Additionally, the expression of genes such as *IL1B*, *TNFAIP6*, *CASP3*, and *NFKB2*, which were upregulated by LPS, was suppressed by RvD1 treatment. In particular, the *BCL-2* gene, known for its role in regulating mitochondrial apoptosis [35] by altering the redox state of mitochondrial sulfhydryl groups and controlling the membrane potential, was significantly upregulated after RvD1 treatment (Figure 5) [36,37]. This finding aligns with previous studies showing that RvD1 modulates anti-inflammatory and anti-apoptotic pathways, notably reducing the activity of the Caspase-3 signaling pathway (Figure 5) [38,39,40]. These results underscore the potential of RvD1 to mitigate LPS-induced inflammatory responses by modulating the Caspase-3 signaling pathway and enhancing BCL-2 expression, thereby offering insights into its therapeutic application for inflammatory conditions [41,42]. This effect was validated at the protein level, revealing that RvD1 treatment markedly inhibited key proteins in the MAPK p38 signaling pathway, including PI3K, MyD88, and NF-κB p65 (Figure 5).

Notably, although the reduced total Caspase-3 observed in this study may suggest a potential anti-apoptotic effect of RvD1, this finding should be interpreted with caution. Cleaved Caspase-3 has been confirmed as a specific marker of apoptosis across different cell types and apoptosis models. A previous study confirmed that RvD1 can upregulate cleaved Caspase-3 in LPS-stimulated bone marrow-derived macrophages (BMDMs), promoting macrophage apoptosis through the Fas/caspase-3 signaling pathway [43]. Therefore, future studies should include apoptosis-specific markers, particularly cleaved Caspase-3, to further clarify the context-dependent effects of RvD1 on apoptotic signaling.

Furthermore, evaluating a range of RvD1 concentrations would help determine its dose-dependent effects on key proteins in the MAPK p38 signaling pathway. To clarify whether NF-κB and MAPK p38 are critical pathways for RvD1 to exert its anti-inflammatory effect, a “forced activation” or “pathway rescue” experimental approach could be utilized [44,45]. For instance, additional groups could be established by supplementing RvD1 treatment with specific activators or inhibitors of NF-κB and p38. Further studies are needed to address the aforementioned issues.

In this study, THP-1 monocytic cells without PMA treatment were chosen as an in vitro cell model for resolvin-mediated inflammation resolution. The viability of THP-1 monocytic cells induced by PMA was reduced, and after treatment with LPS and/or RvD1, the total number of cells was insufficient to meet the minimum requirements for high-throughput RNA sequencing. The 24 h treatment duration was selected based on theoretical predictions and is consistent with Bannenberg’s indication that the optimal duration to promote inflammation resolution should not exceed 48 h [11,15]. Theoretically, prolonged exposure to RvD1 is expected to enhance its effect on promoting inflammation resolution. Therefore, limiting the observation period to 24 h in this study may not fully capture the temporal dynamics of its effect. In Figure 1B, *IL-6* gene expression was significantly increased in the LPS group (*p* = 0.0006), while it was downregulated in the LPS+RvD1 group; however, this reduction was not statistically significant (*p* = 0.1218). This result may be influenced by the duration of treatment in the LPS+RvD1 group, as extended treatment duration has been shown to diminish the magnitude of *IL-6* gene expression downregulation [32]. A possible explanation is that under LPS stimulation, the RvD1 receptor ALX/FPR2 is significantly upregulated at 6 h. Since RvD1 primarily regulates the microRNA expression network through this receptor, it subsequently modulates IL-6 production. Interestingly, compared to the control group, the RvD1 group exhibited a statistically significant upregulation of IL-6 gene expression (*p* = 0.0003). The RvD1-induced elevation of pro-inflammatory factors may be attributed to early signaling events that occur as this pro-resolving mediator initiates the inflammation resolution program. A previous study showed that a 4 h treatment with RvD1 alone induces a transient upregulation of *IL-6* gene expression in mouse peritoneal macrophages [46]. Compared to the LPS group, the gene expression levels of *IL-10* were upregulated in the LPS+RvD1 group; however, this difference was not statistically significant. Prolonging the duration of RvD1 treatment to 48 h might detect a more pronounced effect of RvD1 in inducing an upregulation of *IL-10* gene expression. Compared to the control group, *IL-10* gene expression was significantly increased (*p* = 0.0004) in the RvD1 group. The mechanisms underlying RvD1-mediated upregulation of IL-10 gene expression are multifaceted, primarily involving the activation of intracellular signaling (such as the cAMP pathway) through ALX/FPR2 and potentially enhancing IL-10 gene transcription by activating transcription factors such as CREB or by upregulating specific microRNAs (e.g., miR-208a). These factors also explain the upward trend of *CREB* gene expression levels in the RvD1 group compared to the control group.

In Figure 4, compared to the control group, the protein expression level of PI3K in the RvD1 group showed an upward trend. This may be attributed to the cytoprotective mechanism of RvD1, which is mediated by the activation of the PI3K/Akt pathway. Therefore, when RvD1 is administered to monocytes as a single agent, it upregulates PI3K expression through this signaling cascade. Additionally, compared to the control group, the protein expression of AKT in the LPS group showed a statistically significant increase (* *p* < 0.05). A possible explanation for this finding might be that LPS stimulation did not result in a reduction in substances that subsequently regulate AKT expression, such as Ras kinase, calcium ions, and cytokines. Our mRNA sequencing results suggest that Ras kinase is correlated with the B-Raf serine/threonine kinase (*BRAF*) gene, which is upregulated after LPS treatment (Supplementary Figure S3). Another possible explanation is that THP-1 monocytes secrete specific cytokines to regulate and increase the expression of AKT under LPS stimulation. The most plausible explanation for this observation is that the duration of LPS treatment is too short to downregulate AKT expression.

**Figure 6:**
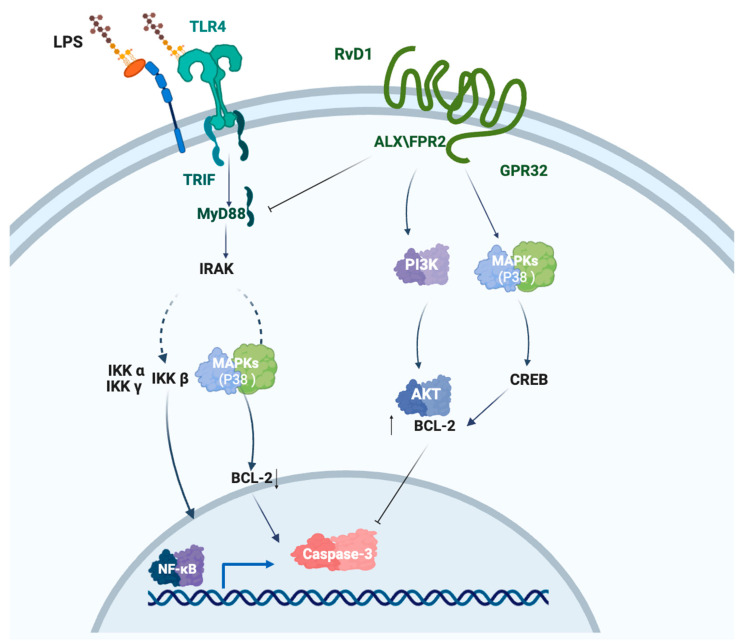
LPS activates the NF-κB and MAPK p38 signaling pathways by binding to TLR4 (created in BioRender). This subsequently activates executioner caspases such as Caspase-3. RvD1, through the ALX/FPR2 receptor, inhibited the MyD88 and PI3K–AKT signaling pathways, promoting the expression of the anti-apoptotic factor BCL-2 and suppressing the expression of Caspase-3. RvD1 also promoted phosphorylation of CREB and upregulated BCL-2 expression through the MAPK p38 signaling pathway (https://BioRender.com/ypqu2di, accessed on 5 March 2026).

Our previous studies have primarily focused on the therapeutic potential of RvD1 in treating periodontitis, demonstrating its potential efficacy in reducing inflammation and promoting tissue regeneration [22,47,48]. In vitro, RvD1 can reduce the expression of hypoxia-induced pro-inflammatory cytokines in periodontal pockets, as well as reverse the effects of hypoxia on the inflammatory phenotype of human periodontal ligament cells (hPDLCs). Furthermore, RvD1 may promote calcium nodule formation in PDLCs by influencing the MAPK p38 signaling pathway through AKT and HIF-1α [22]. In vivo, the RvD1-loaded Gelatin methacrylate effectively alleviated periodontal inflammation and promoted periodontal tissue regeneration in a rat periodontitis model [47]. A recent study demonstrated that RvD1 suppresses the inflammatory response in hPDLCs via the ALX/FPR2 receptor and the TLR4–MyD88–NF-κB/MAPK signaling pathway [48]. This study underscores the dual role of RvD1 in reducing pro-inflammatory responses and enhancing anti-apoptotic mechanisms, primarily through the regulation of the *BCL-2* gene (Figure 6). Upon LPS stimulation, THP-1 cells downregulate MyD88 protein expression levels via the TLR4 signaling pathway. Subsequently, MyD88 serves as a platform to recruit members of the interleukin-1 receptor-associated kinase (IRAK) family, initiating a kinase cascade that leads to NF-κB activation by stimulating the phosphorylation and degradation of the protein IκB. Additionally, with the upregulation of MyD88 protein levels, the activity of the LPS-induced MAPK p38 signaling pathway is enhanced. Furthermore, the downregulation of BCL-2 expression at both gene and protein levels leads to the release of apoptotic execution factors, such as Caspase-3. RvD1, through the ALX/FPR2 receptor, inhibits the LPS-activated MAPK p38 and NF-κB p65 signaling pathways and, by activating the PI3K/Akt signaling pathway, suppresses the expression of Caspase-3 and the downregulation of BCL-2, thereby exerting a negative impact on cell apoptosis. Despite these promising results, this study is limited by the absence of comprehensive high-throughput sequencing and in vivo validation, highlighting the need for further research to elucidate the mechanisms of RvD1 in acute inflammatory responses and its potential therapeutic applications.

## 5. Conclusions

The present study was designed to determine the ability of RvD1 to mediate resolution in human monocytic THP-1 cells in an LPS-induced inflammatory microenvironment. Our findings are consistent with previous studies reporting that RvD1 effectively reduces CASP3 expression and enhances BCL-2 expression, thereby exerting a negative impact on apoptosis. The findings of this study indicate that RvD1 exerts pro-resolving effects by regulating the anti-apoptotic gene *BCL-2* and suppressing the NF-κB p65 and MAPK p38 signaling pathways, and downregulating the gene and protein expression of Caspase-3. These results indicate that RvD1 can attenuate LPS-mediated acute inflammation and demonstrates anti-apoptotic potential. Our study clearly has several limitations. Nevertheless, we believe that our study provides foundational evidence elucidating the mechanism through which RvD1 regulates acute inflammatory responses. Greater efforts are needed in conducting in-depth studies to confirm that RvD1 promotes the resolution of inflammation by regulating apoptosis-related genes.

## Figures and Tables

**Table 1:** The same DEGs were upregulated in the LPS group versus the Control group but downregulated in the LPS+RvD1 group versus the LPS group.

Top20	LPS Group vs. Control Group			Top20	LPS+RvD1 Group vs. LPS Group		
Gene ID	*p* Value	log2Foldchange	Type	Gene ID	*p* Value	log2Foldchange	Type
DNER	0.00006	8.02551	Up	TNFRSF18	0.00041	−7.48294	Down
IL6	0.00002	7.89834	Up	TBC1D3H	0.01602	−7.31492	Down
EDIL3	0.00007	7.66507	Up	SERPINB7	0.00207	−7.09451	Down
TNFRSF18	0.00021	7.48558	Up	IL5RA	0.03219	−6.94365	Down
NEURL3	0.00052	7.21900	Up	CLEC4E	0.00308	−6.90421	Down
SERPINB7	0.00116	7.09701	Up	MAGI1	0.00797	−6.82794	Down
EDAR	0.01557	7.07157	Up	C6orf58	0.04468	−6.74004	Down
PLA1A	0.00114	7.03884	Up	ELOA3	0.00667	−6.66787	Down
IL5RA	0.02059	6.94563	Up	FAM83E	0.01826	−6.47336	Down
CLEC4E	0.00174	6.90748	Up	IDO2	0.02499	−6.21221	Down
MAGI1	0.00496	6.83227	Up	IL33	0.02516	−6.17772	Down
C6orf58	0.02968	6.74201	Up	TLR3	0.03483	−6.10828	Down
ELOA3	0.00401	6.67118	Up	PHEX	0.03283	−6.07108	Down
FAM83E	0.01219	6.47551	Up	TSPAN7	0.03788	−6.00977	Down
IDO2	0.01695	6.21482	Up	XIRP1	0.04106	−5.95996	Down
IL33	0.01698	6.18084	Up	DNER	0.00648	−4.61095	Down
TLR3	0.02451	6.11061	Up	GJB2	0.00401	−4.29684	Down
PHEX	0.02280	6.07392	Up	CCL4	0.00019	−4.26361	Down
TSPAN7	0.02671	6.01252	Up	NEURL3	0.02025	−4.15949	Down
XIRP1	0.02912	5.96346	Up	COL8A1	0.01063	−3.72313	Down
TUBB4A	0.00058	−7.17259	Down	TUBB4A	0.00020	7.54860	Up
C21orf62	0.00064	−7.07809	Down	HSFX1	0.01346	7.36241	Up
MIA	0.00322	−6.91020	Down	LEFTY1	0.00069	7.25846	Up
LEFTY1	0.00165	−6.85777	Down	PRKCG	0.00297	7.16513	Up
SPDYE17	0.02651	−6.75120	Down	NOS2	0.00132	7.09154	Up
NOS2	0.02871	−6.66795	Down	SPDYE17	0.02308	7.05991	Up
HSFX1	0.01556	−6.57859	Down	C21orf62	0.00198	6.93909	Up
TNNI3K	0.03981	−6.47545	Down	ANKRD7	0.00307	6.81678	Up
BTBD11	0.00822	−6.35831	Down	MIA	0.00346	6.79737	Up
ANKRD7	0.00898	−6.35807	Down	BTBD11	0.00941	6.49123	Up
GRM2	0.01062	−6.35806	Down	ANTXRL	0.01359	6.35301	Up
WIPF3	0.00843	−6.34591	Down	FOXI1	0.01320	6.34264	Up
ANTXRL	0.02645	−5.91007	Down	CCR3	0.01419	6.32958	Up
SCN11A	0.03113	−5.84061	Down	WIPF3	0.01893	6.20955	Up
CCR3	0.03135	−5.84014	Down	SCN11A	0.03154	6.01053	Up
PRKCG	0.03938	−5.80499	Down	SNCAIP	0.01841	4.01271	Up
FOXI1	0.03975	−5.74797	Down	CREB3L3	0.02517	3.67301	Up
CREB3L3	0.03453	−3.48527	Down	LDLRAD2	0.00446	3.22943	Up
SNCAIP	0.04818	−3.44535	Down	PLAAT4	0.00746	2.25558	Up
LDLRAD2	0.00170	−2.96918	Down	MYH15	0.03849	2.24643	Up

DESeq2 software (v1.10.1) was used to analyze differentially expressed genes between the experimental and control groups. The type represents the screening result: *p* ≤ 0.05 and |log2(fold change)| ≥ 1 are DEGs; log2(fold change) ≥ 1 is marked as an upregulated gene (Up), and log2(fold change) ≤ −1 is marked as a downregulated gene (Down); those that do not meet the above conditions are not DEGs.

**Table 2:** The DEGs whose expression was upregulated in the LPS group versus the Control group and whose expression was blocked by RvD1 treatment in the LPS+RvD1 group versus the Control group.

Top20	DEGs Were Blocked by RvD1		
Gene ID	log2FoldChange	*p* Value	Type
TNFRSF4	7.76494	0.00012	Up
TNFRSF18	7.48558	0.00021	Up
MBNL2	7.40324	0.00023	Up
TBC1D3H	7.31939	0.00941	Up
NEURL3	7.21900	0.00052	Up
CHGB	7.11993	0.00159	Up
SERPINB7	7.09701	0.00116	Up
EDAR	7.07157	0.01557	Up
PLA1A	7.03884	0.00114	Up
IL5RA	6.94563	0.02059	Up
CASP5	6.92271	0.00252	Up
CLEC4E	6.90748	0.00174	Up
MAGI1	6.83227	0.00496	Up
SGCE	6.81089	0.00265	Up
SLC7A9	6.80532	0.00280	Up
RSPO3	6.76880	0.00370	Up
C6orf58	6.74201	0.02968	Up
ELOA3	6.67118	0.00401	Up
UCHL1	6.66199	0.00443	Up
MISP3	6.65891	0.00427	Up

DESeq2 software was used to analyze DEGs between the experimental and control groups. The type represents the screening result: *p* ≤ 0.05 and |log2(fold change)| ≥ 1 are DEGs; log2(fold change) ≥ 1 is marked as an upregulated gene (top 20), and those that meet the above conditions are DEGs.

**Table 3:** The DEGs are related to inflammation pathways.

Gene ID	Mean. In. Control Group	Mean. In. LPS Group	Mean. In. LPS+RVD1 Group	*p* Value	Type
BCL2L12	3779.53773	3343.41835	3566.69854	0.00498	DEG
CREB1	8258.98700	10,523.92427	9258.53227	0.04415	DEG
IL1B	146.31168	2788.90937	240.56399	0.00078	DEG
TNFAIP6	10.42838	433.68143	43.09317	0.00449	DEG
NFKB2	2357.48480	6125.46109	3900.83018	0.04063	DEG
CASP3	2834.82122	3345.00247	3181.67521	0.03366	DEG

Mean. In. represents the average expression level of DEGs in this group.

## Data Availability

The datasets generated and analyzed in this study are available from the corresponding author. The raw RNA-sequence data supporting the conclusions of this article will be made available by the authors on request.

## Supplementary Materials

The following supporting information can be downloaded at: https://www.mdpi.com/article/10.3390/biomedicines14051124/s1, Figure S1: Principal component analysis. Principal component analysis was performed to visualize the gene expression profiles of three sample groups: the Control group, the LPS group, and the LPS+RvD1 group, which revealed distinct clustering patterns. The first two principal components explained approximately 69.47% and 23.81% of the total variance, respectively; Figure S2: Hierarchical clustering. Each of these mRNAs was grouped into distinct clusters based on their relative abundance. Red represents relatively highly expressed DEGs, and green represents relatively lowly expressed DEGs; Figure S3: Effects of Resolvin D1 on MAPK Signaling in LPS-Treated THP-1 Cells. (A) Differentially expressed mRNA enrichment metabolic diagram: MAPK signaling pathway (LPS group vs. Control group). Red boxes represent upregulated genes in the pathway, and blue boxes represent downregulated genes (each box represents a gene or enzyme, and the green boxes represent species-specific genes or enzymes). (B) Differentially expressed mRNA enrichment metabolic diagram: MAPK signaling pathway (LPS+RvD1 group vs. LPS group); Table S1: Sequences of the primer pairs used in this analysis.

## Abbreviations

The following abbreviations are used in this manuscript:

RvD1	Resolvin D1
LPS	Lipopolysaccharide
IL-1β	Interleukin-1β
TNF-α	Tumor necrosis factor
ELISA	Enzyme-linked immunosorbent assay
mRNA-seq	mRNA sequencing
DEG_S_	Differentially expressed genes
qPCR	Quantitative PCR
WB	Western blotting
IL-6	Interleukin-6
ALX/FPR2	Formyl peptide receptor 2
PMA	Phorbol myristate acetate
PCA	Principal component analysis
CREB	cAMP response element binding protein
NRF2	Nuclear Factor Erythroid 2-Related Factor 2
BCL-2	B-cell lymphoma-2
MyD88	Myeloid differentiation factor 88
NF-κB	Nuclear factor-κB
AKT	Protein kinase B
PI3K	Phosphoinositide 3-Kinase
CASP3	Cysteine-dependent aspartate-specific protease-3
GO	Gene Ontology
BP	Biological processes
CC	Cellular component
TNFAIP6	TNF receptor-associated factor 6
BRAF	B-Raf serine/threonine kinase

## References

[1] Halade G.V., Kain V., Dillion C., Beasley M., Dudenbostel T., Oparil S., Limdi N.A. (2020). Race-based and sex-based differences in bioactive lipid mediators after myocardial infarction. ESC Heart Fail..

[2] Halade G.V., Norris P.C., Kain V., Serhan C.N., Ingle K.A. (2018). Splenic leukocytes define the resolution of inflammation in heart failure. Sci. Signal..

[3] Newton K., Dixit V.M. (2012). Signaling in innate immunity and inflammation. Cold Spring Harb. Perspect. Biol..

[4] Boehncke W., Schön M., Giromolomi G., Bos J., Thestrup-Pedersen K., Cavani A., Nestle F., Bonish B., Campbell J., Nickoloff B. (2005). Leukocyte extravasation as a target for anti-inflammatory therapy-Which molecule to choose?. Exp. Dermatol..

[5] Liu J., Kang R., Tang D. (2024). Lipopolysaccharide delivery systems in innate immunity. Trends Immunol..

[6] Sano M., Uchida T., Igarashi M., Matsuoka T., Kimura M., Koike J., Fujisawa M., Mizukami H., Monma M., Teramura E. (2020). Increase in the Lipopolysaccharide Activity and Accumulation of Gram-Negative Bacteria in the Stomach With Low Acidity. Clin. Transl. Gastroenterol..

[7] Chaiwut R., Kasinrerk W. (2022). Very low concentration of lipopolysaccharide can induce the production of various cytokines and chemokines in human primary monocytes. BMC Res. Notes.

[8] Yu C., York B., Wang S., Feng Q., Xu J., O’Malley B.W. (2007). An essential function of the SRC-3 coactivator in suppression of cytokine mRNA. Mol. Cell.

[9] Crofford L.J. (1997). COX-1 and COX-2 tissue expression: Implications and predictions. J. Rheumatol. Suppl..

[10] Bain C.C., Bravo-Blas A., Scott C.L., Perdiguero E.G., Geissmann F., Henri S., Malissen B., Osborne L.C., Artis D., Mowat A.M. (2014). Constant replenishment from circulating monocytes maintains the macrophage pool in the intestine of adult mice. Nat. Immunol..

[11] Bannenberg G.L., Chiang N., Ariel A., Arita M., Tjonahen E., Gotlinger K.H., Hong S., Serhan C.N. (2005). Molecular circuits of resolution: Formation and actions of resolvins and protectins. J. Immunol..

[12] Chiang N., Fredman G., Bäckhed F., Oh S.F., Vickery T., Schmidt B.A., Serhan C.N. (2012). Infection regulates pro-resolving mediators that lower antibiotic requirements. Nature.

[13] Abdulnour R., Sham H., Douda D., Colas R., Dalli J., Bai Y., Ai X., Serhan C., Levy B. (2016). Aspirin-triggered resolvin D1 is produced during self-resolving gram-negative bacterial pneumonia and regulates host immune responses for the resolution of lung inflammation. Mucosal Immunol..

[14] Rajasagi N.K., Bhela S., Varanasi S.K., Rouse B.T. (2017). Frontline Science: Aspirin-Triggered Resolvin D1 Controls Herpes Simplex Virus-Induced Corneal Immunopathology. J. Leukoc. Biol..

[15] Recchiuti A., Krishnamoorthy S., Fredman G., Chiang N., Serhan C.N. (2011). MicroRNAs in Resolution of Acute Inflammation: Identification of Novel Resolvin D1-Mirna Circuits. FASEB J..

[16] Blaudez F., Ivanovski S., Fournier B., Vaquette C. (2022). The utilisation of resolvins in medicine and tissue Engineering. Acta Biomater..

[17] Liu G.J., Tao T., Wang H., Zhou Y., Gao X., Gao Y.Y., Hang C.H., Li W. (2020). Functions of Resolvin D1-Alx/Fpr2 Receptor Interaction in the Hemoglobin-Induced Microglial Inflammatory Response and Neuronal Injury. J. Neuroinflamm..

[18] Suzuki T., Sato Y., Sano K., Arashiro T., Katano H., Nakajima N., Shimojima M., Kataoka M., Takahashi K., Wada Y. (2020). Severe Fever with Thrombocytopenia Syndrome Virus Targets B Cells in Lethal Human Infections. J. Clin. Investig..

[19] Wu C., Su Z., Lin M., Ou J., Zhao W., Cui J., Wang R.F. (2017). NLRP11 attenuates Toll-like receptor signaling by targeting TRAF6 for degradation via the ubiquitin ligase RNF19A. Nat. Commun..

[20] Wang J.G., Williams J.C., Davis B.K., Jacobson K., Doerschuk C.M., Ting J.P., Mackman N. (2011). Monocytic microparticles activate endothelial cells in an IL-1β-dependent manner. Blood.

[21] Croasdell A., Sime P.J., Phipps R.P. (2016). Resolvin D2 Decreases Tlr4 Expression to Mediate Resolution in Human Monocytes. FASEB J..

[22] Cai J., Liu J., Yan J., Lu X., Wang X., Li S., Mustafa K., Wang H., Xue Y., Mustafa M. (2022). Impact of Resolvin D1 on the inflammatory phenotype of periodontal ligament cell response to hypoxia. J. Periodontal Res..

[23] Mustafa M., Zarrough A., Bolstad A.I., Lygre H., Mustafa K., Hasturk H., Serhan C., Kantarci A., Van Dyke T.E. (2013). Resolvin D1 protects periodontal ligament. Am. J. Physiol.-Cell Physiol..

[24] Vasconcelos D.P., Costa M., Amaral I.F., Barbosa M.A., Águas A.P., Barbosa J.N. (2015). Development of an immunomodulatory biomaterial: Using resolvin D1 to modulate inflammation. Biomaterials.

[25] Mortazavi A., Williams B.A., McCue K., Schaeffer L., Wold B. (2008). Mapping and quantifying mammalian transcriptomes by RNA-Seq. Nat. Methods.

[26] Chan M.M., Moore A.R. (2010). Resolution of inflammation in murine autoimmune arthritis is disrupted by cyclooxygenase-2 inhibition and restored by prostaglandin E2-mediated lipoxin A4 production. J. Immunol..

[27] Buckley C.D., Gilroy D.W., Serhan C.N. (2014). Proresolving Lipid Mediators and Mechanisms in the Resolution of Acute Inflammation. Immunity.

[28] Legler D.F., Bruckner M., Uetz-von Allmen E., Krause P. (2010). Prostaglandin E_2_ at New Glance: Novel Insights in Functional Diversity Offer Therapeutic Chances. Int. J. Biochem. Cell Biol..

[29] Serhan C.N., Chiang N., Van Dyke T.E. (2008). Resolving Inflammation: Dual Anti-Inflammatory and Pro-Resolution Lipid Mediators. Nat. Rev. Immunol..

[30] Recchiuti A. (2013). Resolvin D1 and Its Gpcrs in Resolution Circuits of Inflammation. Prostaglandins Other Lipid Mediat..

[31] Mısırlıoglu N.F., Ergun S., Kucuk S.H., Himmetoglu S., Ozen G.D., Sayili U., Uzun N., Uzun H. (2025). The Importance of Resolvin D1, LXA4, and LTB4 in Patients with Acute Pancreatitis Due to Gallstones. Medicina.

[32] Rey C., Nadjar A., Buaud B., Vaysse C., Aubert A., Pallet V., Layé S., Joffre C. (2016). Resolvin D1 and E1 promote resolution of inflammation in microglial cells in vitro. Brain Behav. Immun..

[33] Wang Y., Tang L. (2015). Multiplexed gold nanorod array biochip for multi-sample analysis. Biosens. Bioelectron..

[34] Birzele F., Schaub J., Rust W., Clemens C., Baum P., Kaufmann H., Weith A., Schulz T.W., Hildebrandt T. (2010). Into the unknown: Expression profiling without genome sequence information in CHO by next generation sequencing. Nucleic Acids Res..

[35] Hikita H., Takehara T., Shimizu S., Kodama T., Li W., Miyagi T., Hosui A., Ishida H., Ohkawa K., Kanto T. (2009). Mcl-1 and Bcl-xL cooperatively maintain integrity of hepatocytes in developing and adult murine liver. Hepatology.

[36] Rahman M.A., Amin A.R., Wang D., Koenig L., Nannapaneni S., Chen Z., Wang Z., Sica G., Deng X., Chen Z. (2023). RRM2 regulates Bcl-2 in head and neck and lung cancers: A potential target for cancer therapy. Clin. Cancer Res..

[37] Manickam D.S., Hirata A., Putt D.A., Lash L.H., Hirata F., Oupický D. (2008). Overexpression of Bcl-2 as a proxy redox stimulus to enhance activity of non-viral redox-responsive delivery vectors. Biomaterials.

[38] Papagiannakopoulos T., Shapiro A., Kosik K.S. (2008). MicroRNA-21 targets a network of key tumor-suppressive pathways in glioblastoma cells. Cancer Res..

[39] Benabdoune H., Rondon E.-P., Shi Q., Fernandes J., Ranger P., Fahmi H., Benderdour M. (2016). The role of resolvin D1 in the regulation of inflammatory and catabolic mediators in osteoarthritis. Inflamm. Res..

[40] Nelson J.W., Leigh N.J., Mellas R.E., McCall A.D., Aguirre A., Baker O.J. (2014). ALX/FPR2 receptor for RvD1 is expressed and functional in salivary glands. Am. J. Physiol.-Cell Physiol..

[41] Maira S.M., Finan P., Garcia-Echeverria C. (2010). From the bench to the bed side: PI3K pathway inhibitors in clinical development. Phosphoinositide 3-Kinase in Health and Disease.

[42] Gocher A.M., Azabdaftari G., Euscher L.M., Dai S., Karacosta L.G., Franke T.F., Edelman A.M. (2017). Akt activation by Ca^2+^/calmodulin-dependent protein kinase 2 (CaMKK_2_) in ovarian cancer cells. J. Biol. Chem..

[43] Xiang S.-Y., Ye Y., Yang Q., Xu H.R., Shen C.-X., Ma M.-Q., Jin S.-W., Mei H.-X., Zheng S.-X., Smith F.-G. (2021). RvD1 accelerates the resolution of inflammation by promoting apoptosis of the recruited macrophages via the ALX/FasL-FasR/caspase-3 signaling pathway. Cell Death Discov..

[44] Xu J., Duan X., Hu F., Poorun D., Liu X., Wang X., Zhang S., Gan L., He M., Zhu K. (2018). Resolvin D1 attenuates imiquimod-induced mice psoriasiform dermatitis through MAPKs and NF-κB pathways. J. Dermatol. Sci..

[45] Li J., Deng X., Bai T., Wang S., Jiang Q., Xu K. (2020). Resolvin D1 mitigates non-alcoholic steatohepatitis by suppressing the TLR4- MyD88-mediated NF-κB and MAPK pathways and activating the Nrf2 pathway in mice. Int. Immunopharmacol..

[46] Kain V., Halade G.V. (2019). Immune responsive resolvin D1 programs peritoneal macrophages and cardiac fibroblast phenotypes in diversified metabolic microenvironment. J. Cell. Physiol..

[47] Yan J., Cai J., Pan X., Li S., Fenton C.G., Fenton K.A., Kantarci A., Xue Y., Xue Y., Xing Z. (2025). Resolvin D1 Modulates the Inflammatory Processes of Human Periodontal Ligament Cells via NF-κB and MAPK Signaling Pathways. Biomedicines.

[48] Xing Z., Liu J., Cai J., Jiang X., Liang J., Fujio M., Hadler-Olsen E., Wang J., Kantarci A., Xue Y. (2024). The Application of Resolvin D1-Loaded Gelatin Methacrylate in a Rat Periodontitis Model. Pharmaceutics.

