# Supplementary material for: Resolvin D1 in the Lipopolysaccharide-Induced Inflammatory Microenvironment Mediates Resolution in Human Monocytic THP-1 Cells
Source: Biomedicines. 2026 May 15;14(5):1124. doi: 10.3390/biomedicines14051124 (PMC13204318; doi:10.3390/biomedicines14051124)
Supplement: Supplementary file 1 [file biomedicines-14-01124-s001.zip › Supplementary Figures (Revision).pdf]

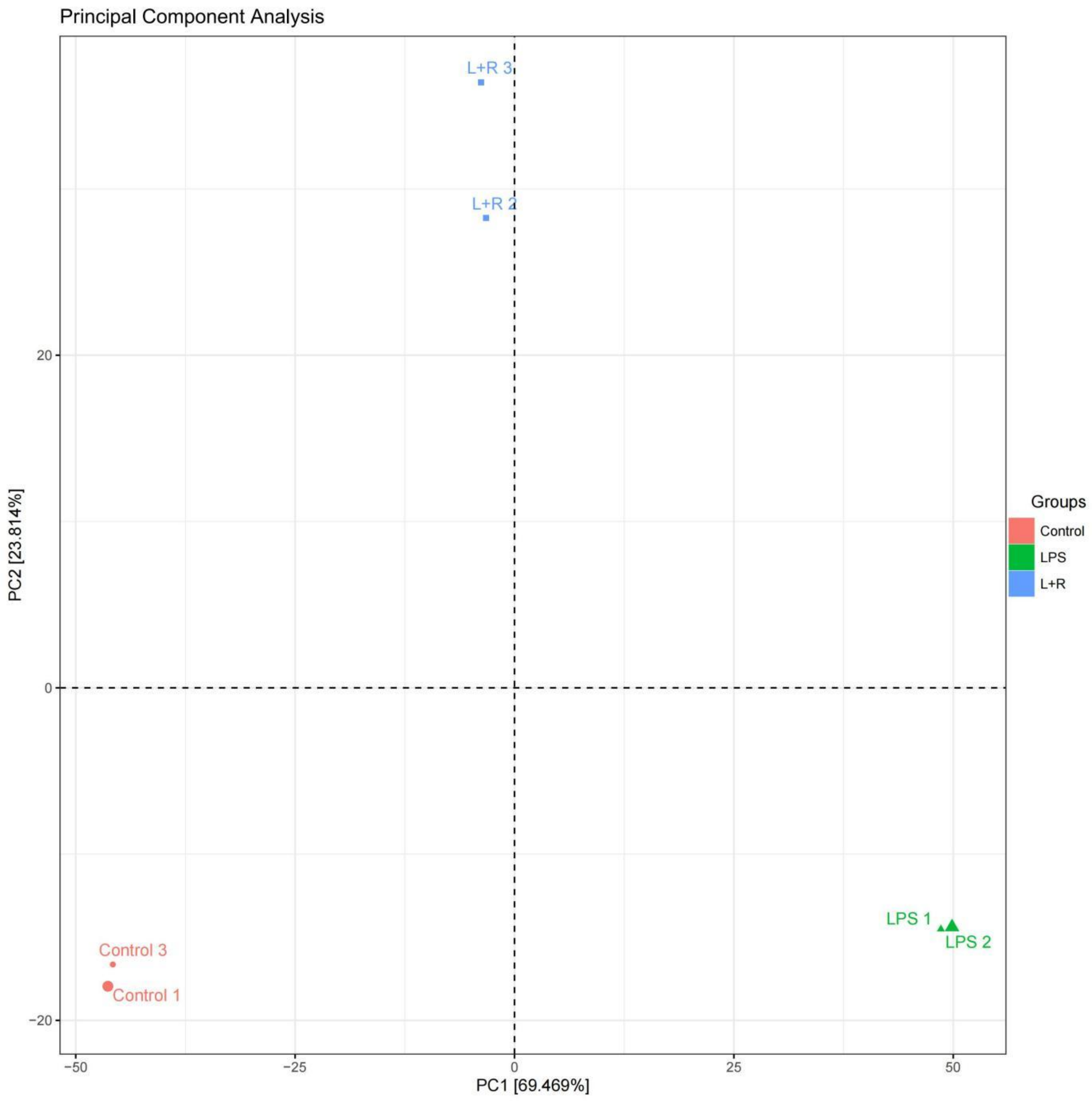

Figure S1 Principal component analysis

Principal component analysis was performed to visualize the gene expression profiles of three sample groups: the Control group, the LPS group, and the LPS+RvD1 group, which revealed distinct clustering patterns. The first two principal components explained approximately 69.47% and 23.81% of the total variance, respectively.

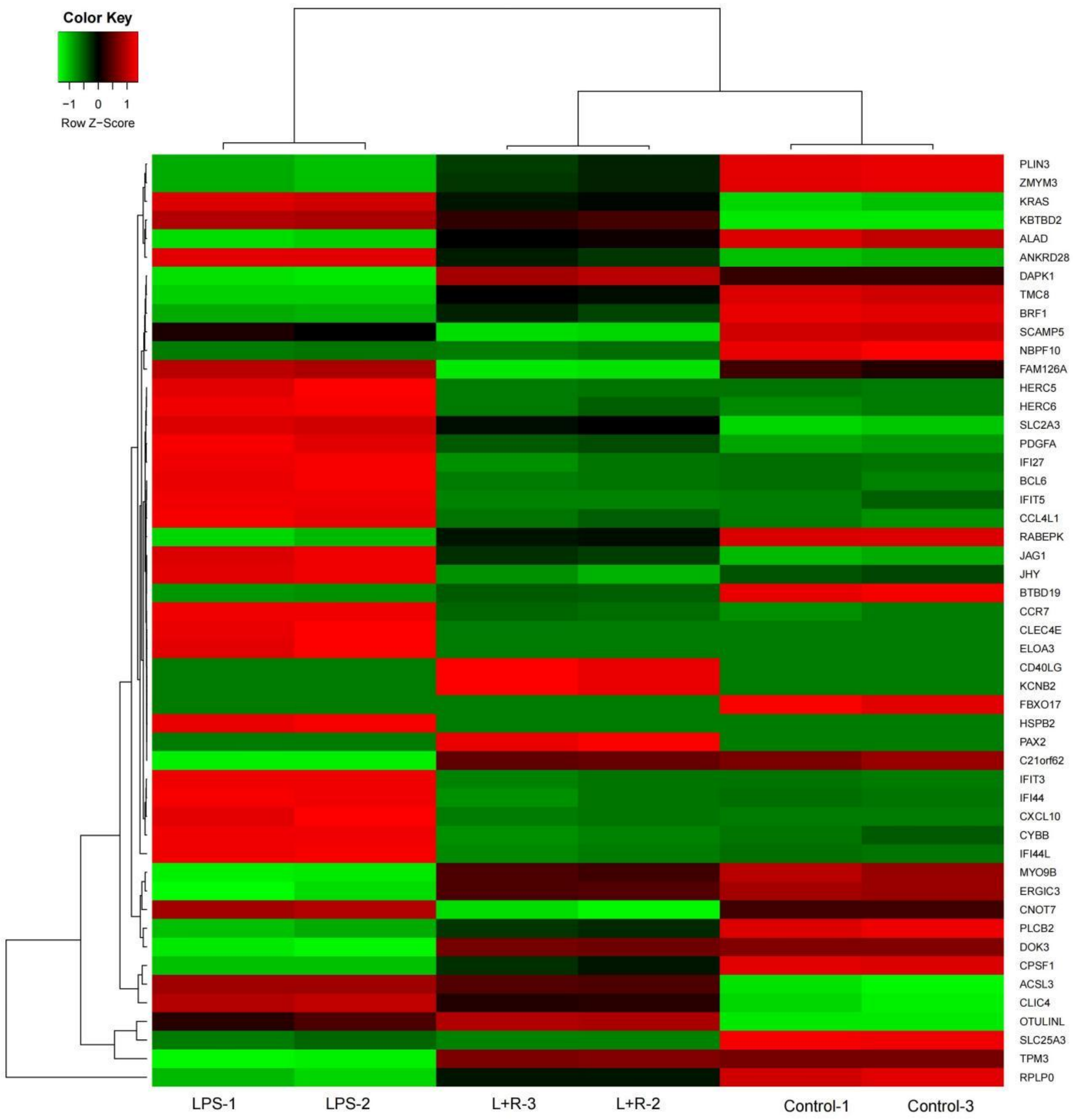

Figure S2 Hierarchical clustering

Each of these mRNAs was grouped into distinct clusters based on their relative abundance. Red represents relatively highly expressed DEGs, and green represents relatively lowly expressed DEGs.

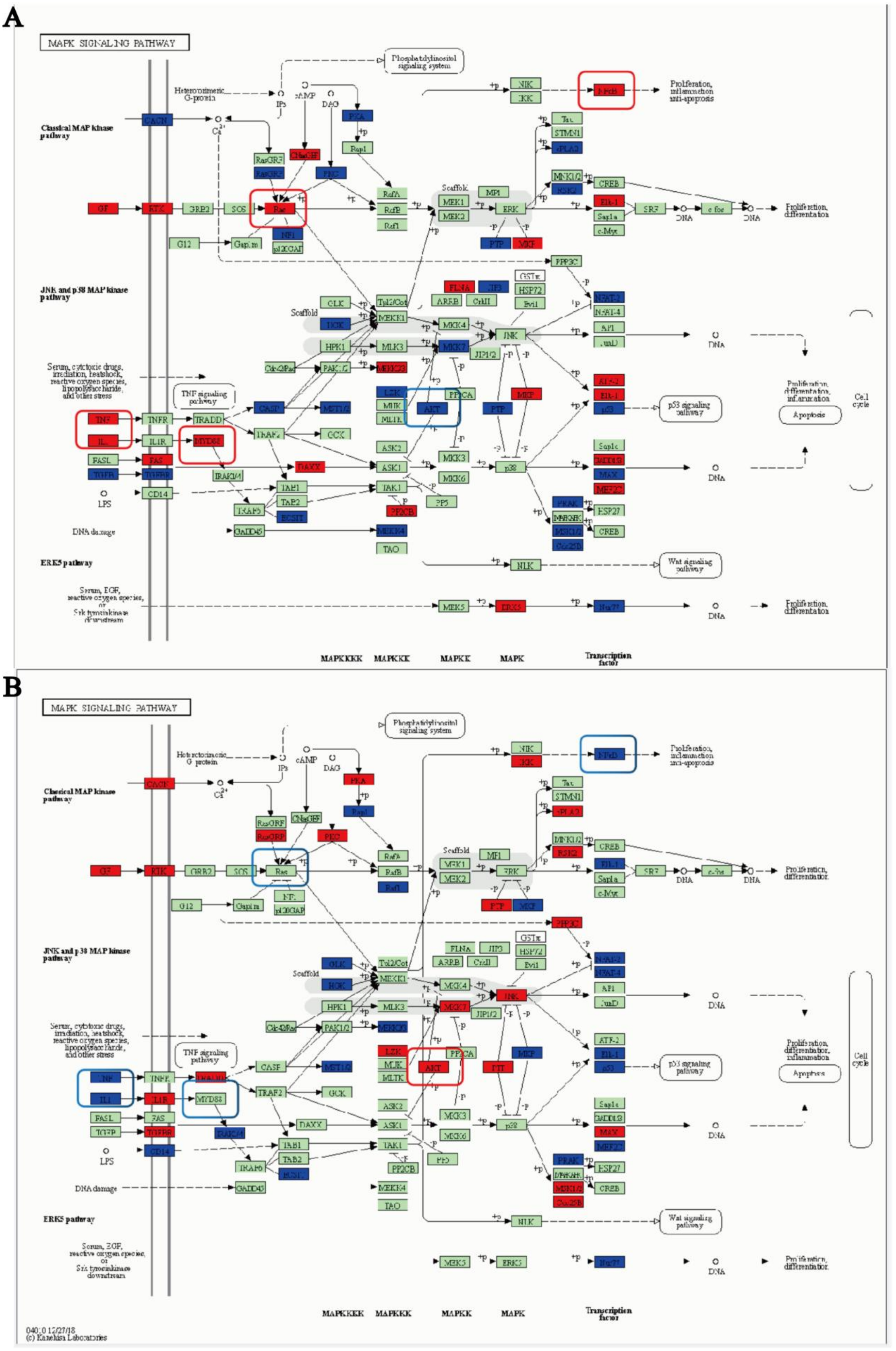

Figure S3. Effects of Resolvin D1 on MAPK Signaling in LPS-Treated THP-1 Cells. (A) Differentially expressed mRNA enrichment metabolic diagram: MAPK signaling pathway (LPS group vs. Control group). Red boxes represent upregulated genes in the pathway, and blue boxes represent downregulated

genes (each box represents a gene or enzyme, and the green boxes represent species-specific genes or enzymes). (B) Differentially expressed mRNA enrichment metabolic diagram: MAPK signaling pathway (LPS+RvD1 group vs. LPS group).
