# Supplementary material for: Resolvin D1 in the Lipopolysaccharide-Induced Inflammatory Microenvironment Mediates Resolution in Human Monocytic THP-1 Cells
Source: Biomedicines. 2026 May 15;14(5):1124. doi: 10.3390/biomedicines14051124 (PMC13204318; doi:10.3390/biomedicines14051124)
Supplement: Supplementary file 1 [file biomedicines-14-01124-s001.zip › Supplementary Table S1.pdf]

Table S1 Sequences of the primer pairs used in this analysis

Gene(human)	Forward Primer sequences (5′-3′)	Reverse Primer sequences (5′-3′)
GAPDH	GCACCGTCAAGGCTGAGAAC	TGGTGAAGACGCCAGTGGA
TNF-α	GTAGCCCATGTTGTAGCAAACCC	TTATCTCTCAGCTCCACGCCA
IL-6	AAGCCAGAGCTGTGCAGATGAGTA	TGGCGAGCTCAGGTACTTCTG
IL-1β	CCAGGGACAGGATATGGAGCA	TTCAACACGCAGGACAGGTACAG
IL-10RB	AACAACCCATGACGAAACGG	AGGGGAGAAGGCGTACTTTG
CREB	GAGCTTGTACCACCGGTAACT	GATACCTGGGCTAATGTGGC
BCL-2	AACATCGCCCTGTGGATGAC	ACAAAGGCATCCCAGCCTC
CAPS3	AGGCGGTTGTAGAAGAGTTTCG	GCTCGCTAACTCCTCACGG
Nrf2	GTGCTGTCAAGGGACATGGA	GCTCATA CTCTTTCCGTCGCT
